# Reply to Francescato et al.: on correct computation of confidence intervals for kinetic parameters

**DOI:** 10.14814/phy2.14181

**Published:** 2019-07-18

**Authors:** Richie P. Goulding, Denise M. Roche, Simon Marwood

**Affiliations:** ^1^ School of Health Sciences Liverpool Hope University Liverpool United Kingdom

We thank Francescato and colleagues for their interest in our studies (Goulding et al., 2018a, 2018b).

Our decision to use the 1‐sec interpolation method was borne out of the data of Benson et al., (2017b). These authors used 2*10^5^ Monte Carlo simulations of moderate‐intensity exercise transitions to determine the impact of various averaging and fitting procedures on $\tau_{\dot{V}O_{2}}$ estimation. A particular strength of this study was the ability to produce a clean $\left[\dot{V}{\text{O}}_{2}\right]$ kinetic trace with known (i.e. “true”) parameters. Subsequently, this trace was sampled using simulations of breathing frequency and the addition of Gaussian noise similar to that associated with experimentally obtained $\left[\dot{V}{\text{O}}_{2}\right]$ data, but with known underlying kinetic parameters. This study, therefore, represents the only study which has allowed precise quantification of both the precision and the accuracy of $\left[\dot{V}{\text{O}}_{2}\right]$ averaging methods.

Benson et al. (2017b) reported a statistically significant difference in the distributions of $\tau_{\dot{V}O_{2}}$ among 1‐sec interpolation vs. binned, stacked, and separate averaging methods; the 1‐sec interpolation was more accurate and displayed narrower variance than all other methods. Hence, the statement by Francescato et al. that “this procedure was not even supported by the results of Benson et al. (2017a), although it was suggested in their abstract (Francescato et al., 2017)” is simply not true. Although pragmatically the $\tau_{\dot{V}O_{2}}$ values derived from the other averaging methods were very similar in this study (Benson et al., 2017b), the conclusion that 1‐sec interpolation provided the most accurate and precise data averaging method is ineluctable on the basis of the data presented by Benson et al., (2017b).

We acknowledge the concerns that Francescato et al. have raised in their letter and previously (Francescato et al., 2014a, 2014b, 2015, 2017) regarding the “cloning” effect of linear interpolation on the 95%_CI_ of $\tau_{\dot{V}O_{2}}$. However, our rationale for employing this method was to choose the data averaging technique that resulted in the most accurate and precise values of $\tau_{\dot{V}O_{2}}$ (i.e. those most reflective of the true underlying kinetics), thus enabling the evaluation of interventions that manipulate $\tau_{\dot{V}O_{2}}$. This consideration was particularly pertinent in the design of our research under discussion (Goulding et al., 2018a, 2018b), and elsewhere (Goulding et al., 2017), requiring the use of single transitions to determine the $\left[\dot{V}{\text{O}}_{2}\right]$ kinetic responses to severe‐intensity exercise, which in turn removed the possibility of performing the stacked averaging method. It is well known that the signal/noise ratio of $\left[\dot{V}{\text{O}}_{2}\right]$ data is lower with that obtained from single transitions as opposed to multiple averaged transitions (Lamarra et al., 1987). Under such conditions it might be expected that the small quantitative differences reported between averaging methods reported by Benson et al., (2017b) may be larger, and hence it was sagacious to err on the side of caution and select the method most likely to provide the most accurate and precise estimates of $\tau_{\dot{V}O_{2}}$ (i.e. 1‐sec interpolation).

We would also like to note, as others have previously (Benson et al., 2017a, 2017b), that the 95%_CI_ may now be somewhat redundant. This is because the Monte Carlo simulations of Benson et al., (2017b) provided the minimally important difference to determine changes in $\tau_{\dot{V}O_{2}}$ during interventional and comparative studies: with single transitions, the minimal difference for transitions from an unloaded baseline was ~8 sec; rising to ~9 sec with transitions from an elevated baseline. In our aforementioned studies (Goulding et al., 2018a, 2018b), the differences in $\tau_{\dot{V}O_{2}}$ between unloaded and elevated baseline conditions were 15 and 14 sec, respectively, exceeding the minimally important difference determined by Benson et al., (2017b). Hence the findings of Benson et al., (2017b) may render the 95%_CI_ inappurtenant in resolving whether an intervention has produced a true change in $\tau_{\dot{V}O_{2}}$.

In conclusion, we acknowledge the concerns of Francescato et al. that the 1‐sec interpolation method may produce a 95%_CI_ that is artifactually narrow due to a “cloning” effect. However, we believe that this observation is essentially unimportant with regard to any conclusions drawn from our studies. Specifically, we employed the 1‐sec interpolation procedure (Goulding et al., 2018a, 2018b) as it has previously been shown to provide the most accurate and precise estimates of $\tau_{\dot{V}O_{2}}$ (Benson et al., 2017b). The differences in $\tau_{\dot{V}O_{2}}$ between each condition in these studies were greater than the recently determined minimally important difference to determine significant changes in $\tau_{\dot{V}O_{2}}$ in intervention studies (Benson et al., (2017b). Hence, these considerations give us confidence that the changes in $\tau_{\dot{V}O_{2}}$ that we reported between conditions reflect true changes in $\tau_{\dot{V}O_{2}}$. More importantly for the present discussion, these considerations also lead us to believe that the points raised by Francescato and colleagues are somewhat moot.
